# Using Visual Analogue Scales in eHealth: Non-Response Effects in a Lifestyle Intervention

**DOI:** 10.2196/jmir.5271

**Published:** 2016-06-22

**Authors:** Tim Kuhlmann, Ulf-Dietrich Reips, Julian Wienert, Sonia Lippke

**Affiliations:** ^1^Research Methods, Assessment & iScienceDepartment of PsychologyUniversity of KonstanzKonstanzGermany; ^2^Institute for Social Medicine and EpidemiologyUniversity of LübeckLübeckGermany; ^3^Jacobs Center on Lifelong Learning and Institutional DevelopmentDepartment of Psychology & MethodsJacobs University BremenBremenGermany

**Keywords:** eHealth, RCT, VAS, dropout, visual analogue scale, Internet science, measurement

## Abstract

**Background:**

Visual analogue scales (VASs) have been shown to be valid measurement instruments and a better alternative to Likert-type scales in Internet-based research, both empirically and theoretically [1,2]. Upsides include more differentiated responses, better measurement level, and less error. Their feasibility and properties in the context of eHealth, however, have not been examined so far.

**Objective:**

The present study examined VASs in the context of a lifestyle study conducted online, measuring the impact of VASs on distributional properties and non-response.

**Method:**

A sample of 446 participants with a mean age of 52.4 years (standard deviation (SD) = 12.1) took part in the study. The study was carried out as a randomized controlled trial, aimed at supporting participants over 8 weeks with an additional follow-up measurement. In addition to the randomized questionnaire, participants were further randomly assigned to either a Likert-type or VAS response scale version of the measures.

**Results:**

Results showed that SDs were lower for items answered via VASs, 2*P* (Y ≥ 47 | n=55, *P*=.5) < .001. Means did not differ across versions. Participants in the VAS version showed lower dropout rates than participants in the Likert version, odds ratio = 0.75, 90% CI (0.58-0.98), *P*=.04. Number of missing values did not differ between questionnaire versions.

**Conclusions:**

The VAS is shown to be a valid instrument in the eHealth context, offering advantages over Likert-type scales. The results of the study provide further support for the use of VASs in Internet-based research, extending the scope to senior samples in the health context.

**Trial Registration:**

Clinicaltrials.gov NCT01909349; https://clinicaltrials.gov/ct2/show/NCT01909349 (Archived by WebCite at http://www.webcitation.org/6h88sLw2Y)

## Introduction

### Internet- and eHealth-Based Health Promotion

Internet-based health promotion targeting health behavior change is a promising approach for health behavior change and aftercare programs [3]. It offers a number of advantages over traditional health promotion methods, most notably, the possibility of taking part anywhere at any time, as long as an Internet-connected device is available. Furthermore, it offers the possibility of tailoring the health promotion to the specific users. Tailored content and feedback can be presented based on demographic characteristics such as gender and age and also based on answers to items presented during the health promotion period or previously assessed behavioral outcomes. Such personalization has been shown to increase the effectiveness of health promotion [3,4]. Initiatives of eHealth may also help in increasing the cost-effectiveness of health care programs, as their running costs are comparably low. The field of Internet-based health promotion is in its early stages and thus, guidelines for designing such programs optimally are sparse. This is of crucial importance because the development of eHealth programs is time consuming and the outcome, for example, less mortality due to a healthier lifestyle, of high value [5].

*Dropout* has been discussed as a frequent phenomenon in the literature on Internet research (eg, [6,7]). Musch and Reips found in an early meta-analysis that median dropout is at 35% [8]. Reips [9] lists several factors that may influence dropout by participants in Internet-based research: incentives, placement of demographic questions (early means less dropout), technology used (server-side programming reduces dropout), attractive website design, trustworthiness of website, offering feedback, information about duration and progress, and so forth. Eysenbach writes “… factors, for example, expectation management before the trial or ‘push factors’ such as reminders by the study team, influence the shape and slope (steepness) of the attrition curve.” (p.4) [6].

The possibility for participants to drop out at any time during Internet-based studies also translates to tailored eHealth programs. The implications are potentially more serious, however. Dropout in a tailored eHealth and online health promotion program not only means missing data for the researcher but also a less effective treatment for the participant. In general, eHealth aims at lifestyle changes to increase the health and lower the risk factors for participants who oftentimes are already at high risk. Dropping out of the program lowers the chances of behavior change and can therefore have serious consequences for the individuals’ health and may sometimes lead to more complications and even an earlier death.

Measures to avoid, reduce, control, or use dropout [9,10] such as the *seriousness check*, *warm-up*, and *high hurdle* techniques to identify participants who just want to take a look out of curiosity (Eysenbach consequentially writes about a “curiosity plateau”, p.4 [6]) are even more important in eHealth programs compared with Internet research. This is especially true because dropout is often higher for subgroups with an increased risk for adverse health events. These are participants with the highest possible gain from adhering to the guidelines and suggestions of the program [11]. Furthermore, *missing data* have severe implications for eHealth as well. Tailoring of the health promotion program is dependent on the data provided by participants. Non-response to certain variables therefore results in fewer tailoring opportunities, thus potentially lowering the effectiveness of the whole program [5].

### Visual Analogue Scales

When designing an online questionnaire, the question of how to design items and which answer options to present is always an issue. In the case of measuring the degree of agreement to a statement, the answer options are traditionally often presented in a Likert-type scale format. On these scales, participants have the option to distinguish between a discrete set of answer options to indicate their agreement. They are widely implemented in questionnaires within the medical and social sciences, for example, when measuring subjective health, personality, intentions, or expectancies. Typically, the response to an item is indicated in 5 to 7 gradations ranging from “strongly disagree” to “strongly agree.” An alternative to Likert-type scales with radio buttons are *visual analogue scales* (VASs). VASs are rating scales in a continuous graphical format. Instead of clicking on a radio button to indicate the amount of agreement to a statement, participants click anywhere on a continuous line that stretches between the 2 anchors.

These scales offer a number of advantages over Likert-type scales with regard to psychometric properties and data analyses. They were first described by Hayes and Patterson [12]. A theoretical advantage over discrete scales, such as radio button scales, often used on the Internet is that answers are not restricted to a certain number of response options, but rather, very fine gradations can be measured. In computer- and Internet-based assessment, each pixel in length of a VAS corresponds to a possible value [2]. Data collected with VASs offer more options for statistical procedures than data collected with categorical scales, for example, transformations, recoding of scores, and more power for goodness-of-fit-tests. Mean ratings for assessed scales have been shown to be equal when comparing the value received from VASs to the one gathered via other answer scales in paper-based studies [13] and Internet-based studies [1].

In a paper-based study, Myles et al [14] and Myles and Urquhart [15] found that data from VASs form a linear scale when assessing mild-to-moderate pain intensity. Furthermore, equal changes in intensity corresponded to equal differences in length on VASs. Reips and Funke [2] found that in Web questionnaire items, VASs fulfill the requirements of measurement on the level of an interval scale. Therefore, differences between ratings on VASs can be interpreted in a meaningful way, and the prerequisites for many statistical procedures are met. Reips and Funke [2] also examined whether the length of the presented VAS had an influence on the quality of the data provided. They investigated 3 different lengths: 50, 200, and 800 pixels. Despite small differences between the different length versions of the scale, all 3 versions provided very similar data of high quality. Hayes et al [16] described the possibility of general VASs to maximize the discrimination between neighboring categories and possibly produce less compressed answers at the extremes depending on the anchors used.

### Visual Analogue Scales and Missing Data

The impact of VASs on dropout and missing values differs between studies. Couper et al [17] reported a higher dropout in VAS questionnaires (8.2%) in comparison to questionnaires with radio button scales (4.4%). Frequency of missing values was also higher in the VAS questionnaire version: 6.7% as compared with 1% in radio button scales. They note, however, that these differences are confounded by the use of Java applets in the VAS questionnaire version, which resulted in longer loading times and could have caused technical difficulties. Funke and Reips [1] found no difference with regard to item non-response in their study on semantic differentials, comparing VASs and 5-point Likert scales. In fact, they observed a trend toward more complete responding for the VAS questionnaire version.

Tucker-Seeley [18] conducted a Web-based experiment on students in grades 4 to 12, where the students had to answer the same question in a Likert or VAS format. The results showed that 71.4% of participants preferred to answer questions in a VAS format. Furthermore, in the VAS questionnaire version, 76% of respondents indicated that they felt they could pick an answer that matched exactly the way they felt, compared with only 51% in the Likert questionnaire version. Given the influence of motivational factors on dropout [19,20], these findings also suggest a possible positive influence of the format on non-response.

### Research Questions and Hypotheses

The main goal of this study was to investigate VASs in the applied context of an eHealth program. We replicated previous research, and expected to find lower standard deviations (SDs) in the VAS questionnaire version [1] but no difference in means between VASs and other scales [1,17]. Furthermore, we hypothesized that the use of VAS would reduce dropout and missing values in comparison to the Likert questionnaire version. Although some research showed the opposite effect [17], more recent studies have found a trend toward less missing data [1]. We argue that the progression in technology has widely eliminated technical difficulties when using VASs, and therefore, a main effect on less dropout and missing data remains, even with (older) cardiac patients who otherwise may not be routinely confronted with computerized VASs.

## Methods

### Likert vs Visual Analogue Scales

**Figure 1:**
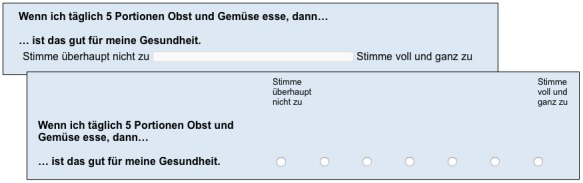
Example item from the questionnaire in VAS and Likert formats.

The experimental manipulation of the present study was the use of either Likert-type scales or VAS scales in the questionnaire at the beginning of the study. The 7-point Likert scale ranged from “Strongly Disagree” to “Strongly Agree,” and answers were indicated via radio buttons. The VAS used the same labels, but a continuous answer scale was presented instead of radio buttons. Both scales were displayed in approximately the same length on the screen, and the number of items per page was identical in both the questionnaire versions. An example item from the Likert scale and VAS scale formats is shown in Figure 1. The manipulation was applied to all items that measured a degree of agreement to a statement. Items with assumed discrete answer options, for example, stages of change, were excluded from the manipulation.

### Characteristics of the eHealth Study

The eHealth program was developed as an aftercare program for cardiac rehabilitation patients. The aim of this program was to provide support for participants in maintaining behaviors learned during cardiac rehabilitation, namely physical activity and a healthy diet. A detailed description of the health promotion program is published in a study protocol [21]. Results investigating the effectiveness of the program were published by Storm et al [22] and Reinwand et al [23]. The program was implemented via a university-external provider of solutions for Internet-based tailored programs. It lasted 8 weeks and took place immediately after the rehabilitation period.The program consisted of an extensive questionnaire at the beginning and end of the period, where behavioral data, social-cognitive variables, and sociodemographic data were assessed. The program was comprised of 8 weekly sessions consisting of different behavior change techniques [21,24]. Depending on indications in the questionnaire and behavioral data during the program, the content was tailored to each participant. Each session was designed to take about 20 minutes to complete. Participants did not have to log in at a specific time but were able to participate when it suited their schedule during the week. Weekly email reminders were sent to every participant (but see 'Dropout across sessions' below.)

### Procedure

**Figure 2:**
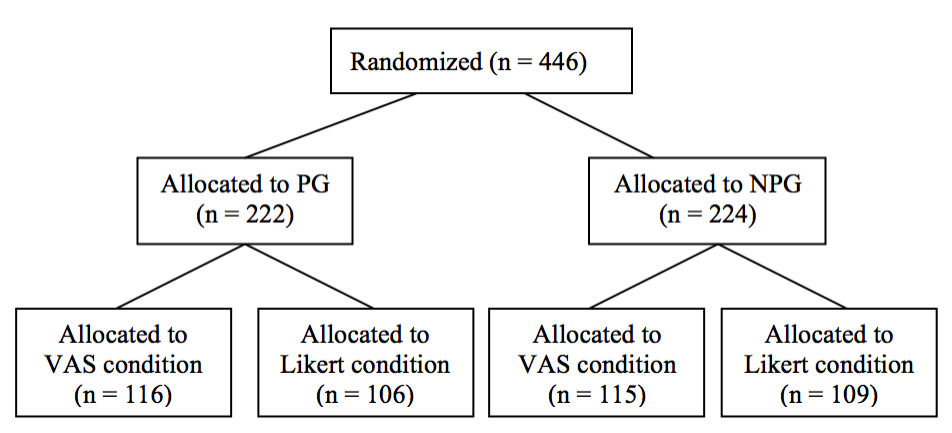
Randomization of participants to groups and response scale versions. NPG: no-program group, PG: program group.

'Recruitment for the study took place at 9 cardiac rehabilitation centers in Northern Germany, via local newspaper articles, research agency online panels and information on Jacobs University’s homepage. Participants interested in joining the eHealth study were asked to create an account on the external provider’s homepage. Creating an account led directly to the baseline questionnaire (*t1*), followed by the first session. Owing to the expected health benefits of the study, as well as providing the control group with an incentive to provide data directly after rehabilitation and after 8 weeks, we decided to make use of a randomized controlled trial (RCT; ClinicalTrials.gov registration number NCT01909349) design with a no-program group (NPG). Participants were randomly assigned to either the program group (PG) or the group that was only asked to fill in the questionnaire (NPG). The study design and the current data, which were analyzed to answer our research questions, are displayed in Figure 2.

In both the groups, participants received questionnaire items either in a 7-point Likert-scale answer format or in a VAS format. This factor was randomly assigned between subjects, with each participant only receiving Likert- or VAS-formatted items. Figure 2 shows the randomization process and flow of participants. The questionnaire at *t1* was the same for the PG and NPG, where the content changed only after the end of the questionnaire. PG participants started with the first session, whereas the NPG received the information that their program would continue 8 weeks later. Six important items for the tailoring were designed as forced choice. However, we designed all other items so they could be skipped without answering because the forced choice format is to be avoided in Internet-based questionnaires for methodological reasons [25,26]. The forced choice items asked for the gender and for a nickname to be chosen for the program. Furthermore, answers were required for 4 items assessing self-efficacy and stages of change.

### Measures

The questionnaire contained constructs typically assessed in health behavior studies. These included stages of change, risk perception, intentions, outcome expectancies, self-efficacy, action plans, coping plans, social support, and habits [21]. Most of the constructs were assessed separately for physical activity and dietary behavior. Furthermore, health-related variables such as height, weight, physical activity, and dietary behavior were also assessed via self-report. Sociodemographic variables included—among others—gender, education, year of birth, native language, and marital status. In total, the questionnaire contained 129 items. Of these, 55 items were presented in either a Likert or VAS format.

Dropout was analyzed separately [20,27]. Furthermore, the dropout across the 8 weekly sessions was analyzed. This analysis could only be performed for the PG, as the NPG was not able to participate in the program until the PG finished the 8-week program, diminishing comparability between the groups.

### Sample Characteristics

The sample consisted of German cardiac patients who took part in ambulatory or stationary rehabilitation and German participants interested in improving their physical activity and diet. The total sample was comprised of 446 participants, about one third (35%) of whom were from the original target group of medical rehabilitation patients. Almost all participants (97%) were native German speakers. The remaining participants indicated English or Lithuanian as their native language. The ages ranged from 24 to 77 years (M=52.4, SD=12.1). Female participants comprised 59% of the sample. The level of education of participants varied, with 52.6% of the sample having finished high school or having earned a higher degree. Most of the participants (75%) were married or in a relationship.

## Results

The results from the analyses of item properties, dropout, and missing values are presented in the following section. In line with Reips and Franek [28], data from participants who dropped out were included in the analyses before the point of their dropout.

### Means and Standard Deviations

Answers in the Likert questionnaire version were recoded to make values comparable to the VAS questionnaire version. Recoded values ranged from 1 to 100. T-tests comparing the mean scores for the 55 items that differed between questionnaire versions were then calculated. There were no differences in the means between the questionnaire versions. In line with previous findings on VASs, SDs were smaller for 47 items in the VAS questionnaire version, 2P (Y ≥ 47 | n=55, *P*=.5) < .001. Items in the VAS questionnaire version had a pooled *SD* of 30, whereas in the Likert questionnaire version, the pooled *SD* was 33. Latent variables were analyzed using structural equation modeling. The 55 items amounted to 15 different measured constructs. For these, confirmatory factor analyses were conducted with free estimates for variance and mean of the latent variables. Scale version was used as a grouping variable in the analyses. In none of the 15 cases was there a mean difference between the latent estimates of the different scale versions (all z's<1.96). The variance of the latent variables was smaller in the VAS group in 13 of the 15 studied constructs. These results are similar to previous findings on distributional properties of VAS scores [1,2].

### Dropout Across Sessions

**Figure 3:**
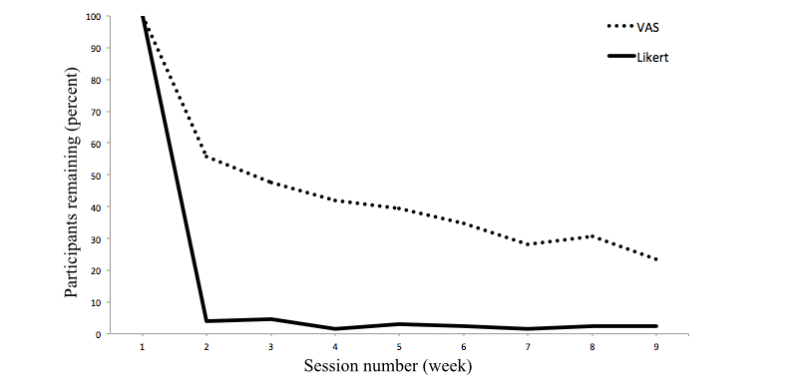
Dropout across the weekly sessions until follow-up.

The analysis of dropout across sessions was only possible in the PG because the NPG was not invited to weekly sessions until the other group ended their treatment. Between-session dropout was compared across the weekly sessions until the follow-up questionnaire was completed. Overall, there were 9 different sessions with 8 weekly parts, and the follow-up questionnaire was administered 1 week later. Because participants were able to continue participation even if a session was missed, the between-session dropout curves do not monotonically decrease. The dropout within the 2 questionnaire versions is shown in Figure 3. There is a sharp drop in the curve for the Likert questionnaire version from session 1 to session 2. Only 5 of the original 106 participants returned for the second session, indicating a dropout of 95% between the 2 sessions. In the VAS questionnaire version, 64 of 116 participants returned for session 2, which equaled a dropout of 53%. The difference was significant, *χ*^2^ (1, *N*=222) = 63.49, *P*<.001, *Φ* = .54.

Because such a large difference in dropout due solely to the answer scale used seemed implausible, further possible differences between the questionnaire versions were examined. After careful examination and queries with the external provider of the survey software, it emerged that participants in the Likert questionnaire version did not receive weekly reminder emails, whereas participants in VAS questionnaire version received weekly reminders. This was due to a technical error without options to restore the defect. Aside from the response scale format, this introduces another difference between questionnaire versions across the weekly sessions. As this confounding factor renders the comparison between groups across the weekly sessions inconclusive, dropout was compared across the time frame in which this factor was not present, namely the responses within, and dropouts from the questionnaire during the first session.

### Dropout During the First Session

**Figure 4:**
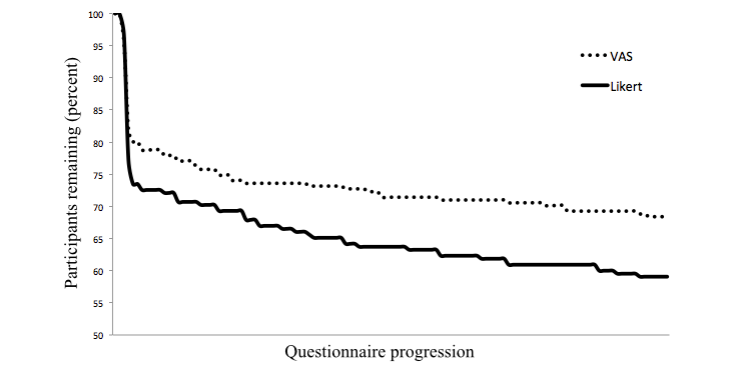
Dropout throughout the entire baseline questionnaire by version.

Dropout was analyzed by determining whether participants were presented a certain item or had already left the questionnaire. Therefore, only skipping a certain page or item was not counted as having dropped out. Figure 4 shows the dropout curves separately for the VAS and Likert questionnaire versions. In the VAS questionnaire version, 73 of 231 initial participants did not finish the questionnaire (31.6%), whereas in the Likert version, 88 of 215 initial participants dropped out (40.9%). A Cox regression with a directed hypothesis resulted in a significant difference between the groups, odds ratio = 0.754, 90% CI (0.58-0.98), *P*=.04, in favor of the VAS scale to the Likert-scale. Neither gender nor the participation in cardiac rehabilitation before participating in the aftercare program was related to dropout.

### Missing Values

The missing data analysis was performed only for items that were presented as either VAS- or Likert-type answer scales. Items of identical format in both the questionnaire versions were excluded.

The total number of items that could be skipped without answering was 55. We analyzed how many presented items were unanswered by participants. In the VAS questionnaire version, 7% (SD=20%) of the presented items were skipped on average, whereas in the Likert-questionnaire version, 8% (SD=18%) of the items were skipped on average. This difference was not significant. Gender differences in skipped items were also not significant. Age of participants was significantly correlated with the mean number of skipped items (*r*=0.26, *P*<.001), indicating that older participants skipped more questions without answering them.

## Discussion

The present study investigated VASs in the applied context of an eHealth program. Lower SDs in the VAS questionnaire version were found for 47 of 55 analyzed items, supporting previous findings that suggest that VAS provide better measurements than Likert scales. There were no differences in mean scores between the VAS and Likert questionnaire versions. Within-session dropout was lower in the VAS questionnaire version. Missing data did not differ between questionnaire versions. Importantly, a technical issue—missing reminder emails in the Likert questionnaire version—was discovered during our investigation and helped identify an important confound, which rendered the between-session dropout analysis inconclusive. The results are discussed in more detail in the following section.

### Distributional Properties of VAS

Findings on the mean scores and SDs replicate findings from previous studies in the eHealth context [1,17]. As most results on eHealth studies so far are based on Likert-type scales, mean differences could possibly hinder comparability of results. The replication of previous findings of equal means in the eHealth context therefore provides an important prerequisite for the implementation of VAS response scales in this area. Smaller SDs when using VASs have also been shown by Funke and Reips [1]. A smaller SD translates into smaller standard errors and thus provides more power to statistical analyses [29]. As the recruitment of participants is always difficult in the health context, this is an important reason to consider VASs in this area.

### Dropout and Missing Values

In line with our hypotheses and with previous findings in the literature, there was a significant lower dropout in the VAS questionnaire version in comparison to the dropout by participants who were provided with Likert-type scales throughout the first session. A trend toward fewer missing values was also observed. Dropout has been argued to reflect lack of motivation to continue participation, among other factors [6,10,20]. Thus, it is conceivable that the lower dropout when implementing VASs is a manifestation of the preference by participants for this type of scale over Likert-type scales or that study participants became more motivated to remain within the study [18].

The implementation of VASs might result in less reactance and an increased interest in continuing with answering the items. These factors potentially increase motivation and thus result in a lower dropout. The effect sizes for both variables at first seem quite small, a 1% difference in missing values and 9.3% difference in dropout; therefore, further research with larger samples is certainly needed to clarify whether the trend observed in our study is stable in the eHealth context. Given the high importance of retaining participants in programs aimed at reducing the risk of life-threatening cardiac incidents, even small differences in dropout have great implications with regard to health. A 9% difference in dropout means that in the VAS condition, 9% more participants receive a program aimed at improving crucial health behaviors to lower the risk of further cardiac incidents.

VASs have been shown in this study and previous research to possess a number of advantages over other answer formats, for example, better distributional properties and lower standard errors [2,14,15]. With less dropout and no increase in missing values, their use in the eHealth context is even more justified than before.Using VASs in a senior sample with low educational status is a true test of their viability in the eHealth context because it ascertains the validity in this context and for a very specific sample that is represented disproportionately highly in eHealth. Showing a positive effect on dropout and no negative effect on missing values, while also replicating previous findings on advantages, is a strong argument for implementing VASs in the eHealth context. Future research should also investigate the possible effect of VASs on adherence to continuing a study.

The impact of answer scales on dropout across sessions could unfortunately not be determined owing to the confounding factor of missing reminder emails in 1 questionnaire version. Future studies are needed to investigate the impact of VASs on dropout across studies. However, the missing reminder problem would have gone unnoticed without our investigation into dropout differences. This additional finding again highlights the importance of reminders (and checking the reminder functionality), and in the case of cardiac patients, this will eventually mean that the lives of several people may be in a better health condition or even prolonged. Dropout and other types of non-response have long been identified as excellent indicators of technical and motivational issues in Internet-based experiments [19,20]. Our present results provide proof that this diagnostic capability of dropout analysis can and should be extended to Internet-based eHealth studies.

### Conclusions

VASs are promising as a viable tool in eHealth studies. Advantages of the format, for example, distributional properties, tend to also hold true in the applied eHealth context. Within-session dropout was lower when VASs were implemented and missing values tended to be lower. Cumulative evidence from several studies points to better measurements with VASs [1,2,14,15]. However, the longer term implications of VASs on between-session dropout will need to be studied, particularly in the context of eHealth.

Care needs to be taken with software design and testing in eHealth programs. It may be better to have the full control that comes with in-house solutions rather than trade in full access for the convenience external providers may suggest.

## Abbreviations

NPG: no-program group
PG: program group
RCT: randomized controlled trial
SD: standard deviation
VAS: visual analogue scale
